# Supplementary material for: Proteome and Interactome Linked to Metabolism, Genetic Information Processing, and Abiotic Stress in Gametophytes of Two Woodferns
Source: Int J Mol Sci. 2023 Aug 4;24(15):12429. doi: 10.3390/ijms241512429 (PMC10419320; doi:10.3390/ijms241512429)
Supplement: Supplementary file 1 [file ijms-24-12429-s001.zip › ijms-2493942-supplementary.pdf]

Table S1. Strongest STRING interactions of proteins extracted from gametophytes of *Dryopteris affinis* and *D. oreades* and classified in the following groups: metabolism of carbohydrates, biosynthesis of amino acids, metabolism of energy and of secondary compounds, transcription and translation, and transport.

Node 1	Node 2	Neighborhood	Gene fusion	Co-occurrence	Co-expression	Experiments	Database	Textmining	Score
**METABOLISM OF CARBOHYDRATES**									
AT2G20420	*AT5G08300*	0.19	0.389	0.448	0.949	0.969	0.949	0.965	0.999
AT2G20420	*AT4G26910*	0.162	0	0	0.916	0.526	0.974	0.714	0.999
AT4G26910	*AT5G08300*	0.189	0.007	0	0.926	0.374	0.972	0.637	0.999
At1g01090	*MAB1*	0	0.69	0.447	0.903	0.942	0.934	0.817	0.999
At1g59900	*MAB1*	0	0.454	0.448	0.903	0.942	0.934	0.823	0.999
PGK1	*TPI*	0.19	0.517	0	0.91	0.419	0.932	0.729	0.999
AT1G54220	*MAB1*	0.173	0.005	0.422	0.838	0.771	0.867	0.776	0.999
**BIOSYNTHESIS OF AMINO ACIDS**									
AT3G23940	*IMS1*	0.19	0	0.238	0.254	0	0.973	0.803	0.997
IIL1	*IMD2*	0.173	0.102	0.369	0.468	0.195	0.973	0.544	0.996
AT1G11860	*SHM3*	0.19	0	0	0.336	0.692	0.932	0.563	0.994
IIL1	*IMS1*	0.185	0	0	0.455	0.214	0.973	0.43	0.993
ATMS1	*MTO3*	0.19	0	0	0.714	0.061	0.884	0.761	0.992
**METABOLISM OF ENERGY**									
AGT	*GOX2*	0	0	0	0.961	0.647	0.966	0.684	0.999
ATPC1	*ATPE*	0.19	0	0	0.908	0.867	0.843	0.615	0.999
ATPC1	*PB*	0.173	0	0.432	0.901	0.905	0.896	0.612	0.999
ATPE	*PB*	0.18	0.15	0	0.985	0.646	0.837	0.966	0.999
ATP1	*ATPC1*	0.19	0	0.395	0.903	0.846	0.834	0.673	0.999
PSAA	*PSAC*	0	0	0.33	0.503	0.995	0.8	0.977	0.999
ATP1	*ATPE*	0.19	0	0	0.906	0.736	0.843	0.916	0.999
ATPA	*ATPC1*	0.19	0	0.421	0.903	0.919	0.834	0.647	0.999
ATPA	*ATPE*	0.19	0	0	0.931	0.736	0.843	0.922	0.999
PSBO2	*PSBP-1*	0	0	0	0.888	0.669	0.36	0.992	0.999
ATPA	*PB*	0.173	0	0.445	0.957	0.905	0.829	0.969	0.999
AT5G08680	*ATPC1*	0.173	0	0.427	0.901	0.818	0.829	0.587	0.999
**METABOLISM OF SECONDARY COMPOUNDS**									
4CL3	*TT5*	0	0	0	0.843	0	0	0.685	0.948
4CL3	*PAL1*	0.08	0	0	0.145	0	0.745	0.763	0.946
4CL3	*PAL4*	0.08	0	0	0.041	0	0.745	0.717	0.928

Node 1	Node 2	Neighborhood	Gene fusion	Co-occurrence	Co-expression	Experiments	Database	Textmining	Score
**METABOLISM OF SECONDARY COMPOUNDS** *(Cont.)*									
PAL1	*PAL4*	0	0	0.449	0.167	0.787	0	0.911	0.819
**TRANSCRIPTION AND TRANSLATION**									
AT1G26880	*AT1G27400*	0.19	0	0	0.986	0.867	0.665	0.415	0.999
AT1G26880	*RPL5B*	0.19	0	0	0.968	0.867	0.667	0.391	0.999
AT1G26880	*AT4G10450*	0.19	0	0	0.982	0.847	0.643	0.536	0.999
AT1G26880	*RPL23AB*	0.19	0	0	0.986	0.867	0.665	0.848	0.999
AT1G26880	*AT2G37190*	0	0	0	0.986	0.847	0.64	0.106	0.999
AT1G26880	*AT1G77940*	0	0	0	0.977	0.82	0.638	0.479	0.999
AT1G26880	*AT2G34480*	0	0	0	0.982	0.82	0.639	0.327	0.999
AT1G26880	*AT3G62870*	0	0	0	0.982	0.867	0.656	0.326	0.999
AT1G26880	*AT3G05560*	0	0	0	0.985	0.847	0.488	0.411	0.999
AT1G26880	*AT2G20450*	0.182	0	0	0.986	0.867	0.665	0.894	0.999
AT1G26880	*AT2G37600*	0	0	0	0.983	0.847	0.651	0.511	0.999
AT1G26880	*AT4G15000*	0.182	0	0	0.985	0.847	0.649	0.577	0.999
AT1G26880	*RPL18*	0	0	0	0.986	0.847	0.648	0.416	0.999
AT1G26880	*AT1G41880*	0	0	0	0.985	0.82	0.638	0.172	0.999
AT1G27400	*AT1G41880*	0	0	0	0.985	0.853	0.661	0.393	0.999
AT1G27400	*RPL18*	0	0	0	0.986	0.867	0.673	0.494	0.999
AT1G27400	*AT4G15000*	0.173	0	0	0.985	0.867	0.673	0.51	0.999
AT1G27400	*AT2G37600*	0	0	0	0.98	0.867	0.673	0.155	0.999
AT1G27400	*AT2G20450*	0.173	0	0	0.986	0.877	0.676	0.599	0.999
AT1G27400	*AT3G05560*	0	0	0	0.985	0.867	0.673	0.516	0.999
AT1G27400	*AT5G58420*	0.19	0	0.385	0.986	0.867	0	0.594	0.999
AT1G27400	*AT3G62870*	0.161	0	0	0.985	0.877	0.676	0.723	0.999
AT1G27400	*AT2G34480*	0	0	0	0.986	0.853	0.661	0.678	0.999
AT1G27400	*AT2G37190*	0.178	0	0.281	0.986	0.867	0.673	0.33	0.999
AT1G27400	*RPL23AB*	0.185	0	0.346	0.986	0.877	0.676	0.598	0.999
AT1G27400	*AT4G36130*	0.171	0	0.319	0.944	0.853	0.661	0.547	0.999
AT1G27400	*AT4G10450*	0.181	0	0.367	0.966	0.867	0.673	0.826	0.999
AT1G27400	*RPL5B*	0.19	0	0	0.977	0.877	0.676	0.546	0.999
AT1G27400	*AT3G52580*	0.181	0	0.292	0.986	0.867	0.143	0.323	0.999
AT1G27400	*AT3G09630*	0.175	0	0.261	0.968	0.877	0.676	0.778	0.999
AT1G41880	*RPL23AB*	0	0	0	0.986	0.853	0.661	0.247	0.999

Node 1	Node 2	Neighborhood	Gene fusion	Co-occurrence	Co-expression	Experiments	Database	Textmining	Score
**TRANSCRIPTION AND TRANSLATION** *(Cont.)*									
AT1G41880	*RPL18*	0	0	0	0.985	0.82	0.645	0.382	0.999
AT1G41880	*AT4G15000*	0	0	0	0.985	0.82	0.645	0.584	0.999
AT1G41880	*AT2G20450*	0	0	0	0.984	0.853	0.661	0.59	0.999
AT1G41880	*AT3G05560*	0	0	0	0.984	0.82	0.645	0.269	0.999
AT1G41880	*AT3G62870*	0	0	0	0.981	0.853	0.661	0.479	0.999
AT1G70600	*RPL5B*	0.19	0	0	0.934	0.867	0.673	0.654	0.999
AT1G70600	*AT3G09630*	0.175	0	0	0.92	0.867	0.904	0.507	0.999
AT1G70600	*RPL23AB*	0.185	0	0	0.943	0.867	0.904	0.512	0.999
AT1G70600	*AT4G36130*	0.171	0	0	0.94	0.82	0.896	0.584	0.999
AT1G70600	*AT4G10450*	0.181	0	0	0.933	0.847	0.899	0.464	0.999
AT1G70600	*AT5G15200*	0.185	0	0	0.944	0.847	0.72	0.588	0.999
AT1G70600	*AT3G05560*	0	0	0	0.943	0.847	0.899	0.517	0.999
AT1G70600	*AT3G62870*	0.065	0	0	0.94	0.867	0.904	0.323	0.999
AT1G70600	*AT2G34480*	0	0	0	0.938	0.82	0.896	0.643	0.999
AT1G70600	*AT4G15000*	0.173	0	0	0.945	0.847	0.899	0.321	0.999
AT1G77940	*AT4G15000*	0	0	0	0.982	0.82	0.645	0.543	0.999
AT1G77940	*RPL18*	0.19	0	0	0.982	0.82	0.645	0.508	0.999
AT1G77940	*AT3G62870*	0	0	0	0.961	0.853	0.661	0.592	0.999
AT1G77940	*AT3G05560*	0	0	0	0.982	0.82	0.645	0.566	0.999
AT1G77940	*AT2G20450*	0	0	0	0.974	0.853	0.661	0.507	0.999
AT1G77940	*RPL23AB*	0	0	0	0.986	0.853	0.661	0.108	0.999
AT2G09990	*AT4G34670*	0	0	0	0.929	0.867	0.965	0.46	0.999
AT2G09990	*AT4G15000*	0.142	0	0	0.954	0.847	0.796	0.405	0.999
AT2G09990	*AT5G28060*	0	0	0	0.934	0.867	0.965	0.584	0.999
AT2G09990	*AT3G05560*	0	0	0	0.951	0.847	0.829	0.586	0.999
AT2G09990	*AT5G15200*	0.179	0	0	0.968	0.847	0.964	0.369	0.999
AT2G09990	*AT4G25740*	0	0	0	0.937	0.847	0.964	0.416	0.999
AT2G09990	*AT2G34480*	0	0	0	0.965	0.82	0.822	0.347	0.999
AT2G09990	*AT5G58420*	0	0	0	0.934	0.847	0.964	0.59	0.999
AT2G09990	*AT3G57490*	0.142	0	0	0.85	0.729	0.958	0.494	0.999
AT2G09990	*AT4G10450*	0.175	0	0	0.93	0.847	0.821	0.494	0.999
AT2G09990	*AT4G30800*	0.172	0	0	0.911	0.847	0.964	0.512	0.999
AT2G09990	*AT5G59240*	0	0	0	0.908	0.867	0.965	0.613	0.999

Node 1	Node 2	Neighborhood	Gene fusion	Co-occurrence	Co-expression	Experiments	Database	Textmining	Score
				**TRANSCRIPTION AND TRANSLATION** *(Cont.)*					
AT2G20450	*AT4G15000*	0	0	0	0.986	0.867	0.673	0.601	0.999
AT2G20450	*AT2G37600*	0	0	0	0.982	0.867	0.673	0.599	0.999
AT2G20450	*RPL18*	0	0	0	0.982	0.867	0.673	0.555	0.999
AT2G20450	*AT3G05560*	0	0	0	0.986	0.867	0.673	0.602	0.999
AT2G20450	*AT5G58420*	0.176	0	0	0.985	0.867	0	0.464	0.999
AT2G20450	*AT3G62870*	0.059	0	0	0.949	0.877	0.676	0.592	0.999
AT2G20450	*AT2G34480*	0	0	0	0.967	0.853	0.661	0.591	0.999
AT2G20450	*AT2G37190*	0	0	0	0.968	0.867	0.673	0.512	0.999
AT2G20450	*RPL23AB*	0.177	0	0	0.986	0.877	0.676	0.835	0.999
AT2G20450	*AT4G10450*	0.173	0	0	0.985	0.867	0.673	0.845	0.999
AT2G20450	*RPL5B*	0.189	0	0	0.96	0.877	0.676	0.637	0.999
AT2G20450	*AT3G09630*	0.142	0	0	0.943	0.877	0.676	0.609	0.999
AT2G34480	*AT3G05560*	0	0	0	0.965	0.82	0.896	0.632	0.999
AT2G34480	*AT5G15200*	0	0	0	0.985	0.82	0.72	0.059	0.999
AT2G34480	*AT3G62870*	0	0	0	0.986	0.853	0.901	0.696	0.999
AT2G34480	*AT4G34670*	0	0	0	0.977	0.853	0.72	0.056	0.999
AT2G34480	*RPL18*	0	0	0	0.985	0.82	0.645	0.225	0.999
AT2G34480	*AT4G15000*	0	0	0	0.985	0.82	0.896	0.244	0.999
AT2G34480	*AT3G09630*	0	0	0	0.982	0.853	0.901	0.718	0.999
AT2G34480	*RPL23AB*	0	0	0	0.969	0.853	0.901	0.477	0.999
AT2G34480	*AT2G37190*	0.182	0	0	0.982	0.82	0.645	0.352	0.999
AT2G34480	*AT4G10450*	0	0	0	0.938	0.82	0.896	0.734	0.999
AT2G37190	*AT4G10450*	0.178	0	0.241	0.951	0.847	0.656	0.569	0.999
AT2G37190	*RPL23AB*	0.182	0	0.203	0.982	0.867	0.673	0.517	0.999
AT2G37190	*AT3G09630*	0.172	0	0	0.944	0.867	0.673	0.595	0.999
AT2G37190	*RPL5B*	0.19	0	0	0.968	0.867	0.673	0.6	0.999
AT2G37190	*RPL18*	0	0	0	0.982	0.847	0.656	0.383	0.999
AT2G37190	*AT4G15000*	0	0	0	0.985	0.847	0.656	0.544	0.999
AT2G37190	*AT3G62870*	0.059	0	0	0.985	0.867	0.673	0.61	0.999
AT2G37190	*AT3G05560*	0	0	0	0.984	0.847	0.656	0.594	0.999
AT2G37190	*AT5G28060*	0	0	0.224	0.982	0.867	0	0.564	0.999
AT2G37600	*RPL23AB*	0	0	0	0.982	0.867	0.673	0.595	0.999
AT2G37600	*AT4G10450*	0	0	0	0.982	0.847	0.656	0.764	0.999

Node 1	Node 2	Neighborhood	Gene fusion	Co-occurrence	Co-expression	Experiments	Database	Textmining	Score
**TRANSCRIPTION AND TRANSLATION** *(Cont.)*									
AT2G37600	*AT4G15000*	0	0	0	0.982	0.847	0.656	0.595	0.999
AT2G37600	*RPL18*	0	0	0	0.966	0.847	0.656	0.835	0.999
AT2G43030	*RPS9*	0.19	0	0.242	0.982	0.876	0	0.544	0.999
AT2G43030	*AT4G01310*	0.185	0	0.311	0.986	0.898	0.424	0.287	0.999
AT3G05560	*AT4G15000*	0	0	0	0.986	0.847	0.899	0.896	0.999
AT3G05560	*RPL18*	0	0	0	0.975	0.847	0.656	0.574	0.999
AT3G05560	*AT3G62870*	0	0	0	0.982	0.867	0.904	0.308	0.999
AT3G05560	*AT5G15200*	0	0	0	0.976	0.847	0.72	0.596	0.999
AT3G05560	*AT5G28060*	0	0	0	0.95	0.867	0.72	0.585	0.999
AT3G05560	*AT4G10450*	0	0	0	0.982	0.847	0.899	0.588	0.999
AT3G05560	*AT4G36130*	0	0	0	0.938	0.82	0.896	0.33	0.999
AT3G05560	*RPL23AB*	0	0	0	0.986	0.867	0.904	0.727	0.999
AT3G05560	*AT3G09630*	0	0	0	0.961	0.867	0.904	0.607	0.999
AT3G05560	*RPL5B*	0	0	0	0.968	0.867	0.673	0.615	0.999
AT3G09630	*RPL18*	0.184	0	0	0.985	0.867	0.673	0.598	0.999
AT3G09630	*RPL23AB*	0.179	0	0.228	0.936	0.877	0.905	0.383	0.999
AT3G09630	*AT4G36130*	0.121	0	0.234	0.939	0.853	0.901	0.602	0.999
AT3G09630	*AT4G10450*	0.175	0	0.248	0.934	0.867	0.904	0.819	0.999
AT3G09630	*RPL5B*	0.19	0	0	0.955	0.877	0.676	0.653	0.999
AT3G09630	*AT4G34670*	0	0	0.345	0.957	0.877	0.838	0.602	0.999
AT3G09630	*AT4G15000*	0.142	0	0	0.95	0.867	0.904	0.509	0.999
AT3G09630	*AT5G15200*	0.179	0	0	0.968	0.867	0.822	0.587	0.999
AT3G09630	*AT5G28060*	0	0	0	0.925	0.877	0.838	0.516	0.999
AT3G09630	*AT3G62870*	0.121	0	0	0.976	0.877	0.905	0.838	0.999
AT3G09630	*AT5G58420*	0	0	0.391	0.982	0.867	0.419	0.587	0.999
AT3G52580	*PFL*	0.181	0	0	0.979	0.774	0.638	0.517	0.999
AT3G52580	*RPS20A*	0.185	0	0	0.984	0.82	0.642	0.585	0.999
AT3G52580	*AT4G15000*	0.173	0	0	0.982	0.847	0.254	0.512	0.999
AT3G52580	*AT5G15200*	0.185	0	0	0.954	0.847	0.654	0.598	0.999
AT3G52580	*AT4G25740*	0	0	0	0.96	0.847	0.655	0.596	0.999
AT3G52580	*AT5G58420*	0	0	0.291	0.982	0.847	0.654	0.417	0.999
AT3G52580	*RPL23AB*	0.185	0	0.273	0.984	0.867	0.257	0.417	0.999
AT3G57490	*AT4G34670*	0	0	0.304	0.912	0.785	0.962	0.355	0.999

Node 1	Node 2	Neighborhood	Gene fusion	Co-occurrence	Co-expression	Experiments	Database	Textmining	Score
**TRANSCRIPTION AND TRANSLATION** *(Cont.)*									
AT3G57490	*AT5G58420*	0.182	0	0.331	0.903	0.729	0.958	0.476	0.999
AT3G57490	*AT4G25740*	0	0	0	0.859	0.729	0.958	0.496	0.999
AT3G57490	*AT5G15200*	0.177	0	0	0.85	0.729	0.958	0.476	0.999
AT3G57490	*AT5G28060*	0	0	0	0.908	0.785	0.962	0.518	0.999
AT3G57490	*AT5G59240*	0	0	0	0.912	0.785	0.962	0.558	0.999
AT3G57490	*AT4G30800*	0.162	0	0	0.893	0.729	0.958	0.489	0.999
AT3G62870	*RPL18*	0.149	0	0	0.986	0.867	0.673	0.547	0.999
AT3G62870	*RPL23AB*	0.175	0	0	0.973	0.877	0.905	0.326	0.999
AT3G62870	*AT5G28060*	0	0	0	0.951	0.877	0.835	0.593	0.999
AT3G62870	*AT5G15200*	0.174	0	0	0.984	0.867	0.821	0.163	0.999
AT3G62870	*AT5G58420*	0	0	0	0.985	0.867	0.394	0.582	0.999
AT3G62870	*AT4G34670*	0	0	0	0.984	0.877	0.835	0.604	0.999
AT3G62870	*AT4G15000*	0.059	0	0	0.973	0.867	0.904	0.367	0.999
AT3G62870	*RPL5B*	0.131	0	0	0.964	0.877	0.676	0.646	0.999
AT3G62870	*AT4G10450*	0.078	0	0	0.941	0.867	0.904	0.644	0.999
AT3G62870	*AT4G36130*	0.104	0	0	0.941	0.853	0.901	0.518	0.999
AT4G01310	*RPS9*	0.19	0	0.338	0.986	0.883	0	0.755	0.999
AT4G10450	*RPL23AB*	0.185	0	0.351	0.985	0.867	0.904	0.49	0.999
AT4G10450	*AT5G15200*	0.185	0	0	0.949	0.847	0.72	0.595	0.999
AT4G10450	*AT5G28060*	0	0	0	0.955	0.867	0.72	0.568	0.999
AT4G10450	*AT4G15000*	0.173	0	0	0.983	0.847	0.899	0.521	0.999
AT4G10450	*RPL5B*	0.19	0	0	0.949	0.867	0.673	0.559	0.999
AT4G10450	*AT4G36130*	0.171	0	0.315	0.925	0.82	0.896	0.544	0.999
AT4G15000	*RPL18*	0	0	0	0.985	0.847	0.656	0.596	0.999
AT4G15000	*RPL23AB*	0.177	0	0	0.986	0.867	0.904	0.581	0.999
AT4G15000	*RPL5B*	0.189	0	0	0.968	0.867	0.673	0.602	0.999
AT4G15000	*AT4G36130*	0.109	0	0	0.937	0.82	0.896	0.414	0.999
AT4G15000	*AT5G28060*	0	0	0	0.982	0.867	0.72	0.609	0.999
AT4G15000	*AT5G15200*	0.177	0	0	0.983	0.847	0.72	0.515	0.999
AT4G15000	*AT4G34670*	0	0	0	0.944	0.867	0.72	0.601	0.999
AT4G25740	*PFL*	0	0	0	0.952	0.774	0.638	0.787	0.999
AT4G25740	*AT4G34670*	0	0	0	0.928	0.867	0.965	0.613	0.999
AT4G25740	*AT5G15200*	0	0	0	0.949	0.847	0.964	0.655	0.999

Node 1	Node 2	Neighborhood	Gene fusion	Co-occurrence	Co-expression	Experiments	Database	Textmining	Score
**TRANSCRIPTION AND TRANSLATION** *(Cont.)*									
AT4G25740	*AT5G28060*	0	0	0	0.928	0.867	0.965	0.856	0.999
AT4G25740	*AT5G58420*	0	0	0	0.94	0.847	0.964	0.462	0.999
AT4G25740	*AT4G30800*	0	0	0	0.926	0.847	0.964	0.592	0.999
AT4G25740	*AT5G59240*	0	0	0	0.91	0.867	0.965	0.602	0.999
AT4G30800	*AT5G59240*	0	0	0	0.926	0.867	0.965	0.605	0.999
AT4G30800	*AT5G15200*	0.182	0	0.223	0.926	0.847	0.964	0.486	0.999
AT4G30800	*AT5G28060*	0	0	0	0.92	0.867	0.965	0.6	0.999
AT4G30800	*AT5G58420*	0.187	0	0.321	0.939	0.847	0.964	0.349	0.999
AT4G30800	*AT4G34670*	0	0	0	0.916	0.867	0.965	0.614	0.999
AT4G34670	*RPL23AB*	0	0	0.239	0.934	0.877	0.72	0.544	0.999
AT4G34670	*AT5G58420*	0	0	0.375	0.959	0.867	0.965	0.545	0.999
AT4G34670	*AT5G28060*	0.059	0	0	0.947	0.877	0.966	0.602	0.999
AT4G34670	*AT5G15200*	0	0	0	0.968	0.867	0.965	0.592	0.999
AT4G34670	*AT5G59240*	0	0	0.223	0.907	0.877	0.966	0.661	0.999
AT4G36130	*RPL23AB*	0.175	0	0.281	0.931	0.853	0.901	0.497	0.999
AT5G15200	*PFL*	0.185	0	0	0.979	0.774	0.638	0.507	0.999
AT5G15200	*RPL18*	0.19	0	0	0.986	0.847	0	0.547	0.999
AT5G15200	*AT5G28060*	0	0	0	0.955	0.867	0.965	0.155	0.999
AT5G15200	*AT5G58420*	0	0	0	0.963	0.847	0.964	0.68	0.999
AT5G15200	*AT5G59240*	0	0	0	0.908	0.867	0.965	0.589	0.999
AT5G28060	*PFL*	0	0	0	0.982	0.841	0.652	0.589	0.999
AT5G28060	*RPS20A*	0	0	0	0.968	0.853	0.661	0.592	0.999
AT5G28060	*RPL23AB*	0	0	0	0.972	0.877	0.72	0.712	0.999
AT5G28060	*AT5G58420*	0	0	0	0.962	0.867	0.965	0.509	0.999
AT5G28060	*AT5G59240*	0	0	0	0.906	0.877	0.966	0.668	0.999
AT5G58420	*PFL*	0	0	0	0.983	0.774	0.638	0.677	0.999
AT5G58420	*RPL23AB*	0.19	0	0.309	0.986	0.867	0	0.417	0.999
AT5G58420	*AT5G59240*	0	0	0.257	0.919	0.867	0.965	0.59	0.999
PFL	*RPL23AB*	0.185	0	0	0.984	0.841	0	0.691	0.999
RPL18	*RPL23AB*	0	0	0	0.983	0.867	0.673	0.355	0.999
RPL18	*RPL5B*	0	0	0	0.982	0.867	0.673	0.597	0.999
RPL23AB	*RPL5B*	0.19	0	0	0.968	0.877	0.676	0.629	0.999

Node 1	Node 2	Neighborhood	Gene fusion	Co-occurrence	Co-expression	Experiments	Database	Textmining	Score
**TRANSPORT**									
AT1G62020	*AT5G05010*	0	0	0	0.921	0.969	0.846	0.73	0.999
AT1G62020	*AT4G34450*	0	0	0	0.928	0.874	0.844	0.671	0.999
AT4G34450	*AT5G05010*	0	0	0	0.922	0.886	0.846	0.73	0.999
